# ST-Elevation Myocardial Infarction (STEMI) in a Morphologically Pediatric Adult With Seckel Syndrome: A Report of a Rare Case

**DOI:** 10.7759/cureus.93458

**Published:** 2025-09-29

**Authors:** Abdulrahman G Qasem, Omar Farooq Al-Nahhas, Mahra A Albeshr, Tamer Al Shouha

**Affiliations:** 1 Emergency Medicine, Tawam Hospital, Al Ain, ARE; 2 Emergency Medicine, Seha - Hospitals, Al Ain, ARE

**Keywords:** conservative management, morphologically pediatric adult, pediatric interventional cardiology, primordial dwarfism, seckel syndrome, st-elevation myocardial infarction

## Abstract

Seckel syndrome is a rare genetic disorder characterized by severe growth retardation, microcephaly, and distinctive craniofacial features. Although cardiovascular anomalies have been sporadically reported, ST-elevation myocardial infarction (STEMI) has not been documented in this population.

To the best of our knowledge, we present the first case of anterior STEMI in a 30-year-old male with Seckel syndrome. The patient, weighing 11.5 kg, presented with acute chest and back pain and was diagnosed with anteroseptal STEMI. His complex medical history, including a coiled intracranial aneurysm, precluded thrombolysis, while his small body size and the absence of pediatric-compatible equipment rendered percutaneous coronary intervention (PCI) unfeasible. Despite maximal intensive care support, the patient developed multiorgan failure and died.

This case highlights the expanding cardiovascular manifestations of Seckel syndrome and the difficulties in applying standard STEMI management protocols to morphologically pediatric adults. The lack of specialized interventional tools and infrastructure in many regions further complicates care. This underscores the urgent need for multidisciplinary care models and specialized pediatric interventional cardiology services for syndromic adults.

## Introduction

Seckel syndrome is an exceptionally rare autosomal recessive disorder characterized by primordial growth retardation, microcephaly, intellectual disability, and distinctive craniofacial features, such as a beaked nose and receding forehead, that have led to its colloquial description as “bird-headed dwarfism” [1]. While the phenotypic and neurodevelopmental characteristics of Seckel syndrome are well documented, its cardiovascular manifestations remain largely underexplored. Reported cardiac anomalies include atrial septal defects and conduction abnormalities, though these are uncommon [2]. A limited number of case reports have described complications such as arrhythmias and complete heart block [1,3]. Moreover, syndromes within the primordial dwarfism spectrum, such as microcephalic osteodysplastic primordial dwarfism type II (MOPD II), have been associated with early-onset coronary and systemic vascular disease, as well as congenital heart defects, arterial stenosis, and cerebrovascular abnormalities [4]. However, to date, ST-elevation myocardial infarction (STEMI) has not been documented in individuals with Seckel syndrome.

Here, we present, to the best of our knowledge, the first reported case of extensive anterior STEMI in a patient with Seckel syndrome, highlighting the unique diagnostic and therapeutic challenges encountered in managing acute coronary syndrome in morphologically pediatric adults with syndromic conditions. Morphologically pediatric adults are individuals who, due to growth disorders such as primordial dwarfism, retain child-sized body proportions despite chronological age [5]. Verbal consent for publication was obtained from the patient’s family prior to documentation of this case.

## Case presentation

**Figure 1:**
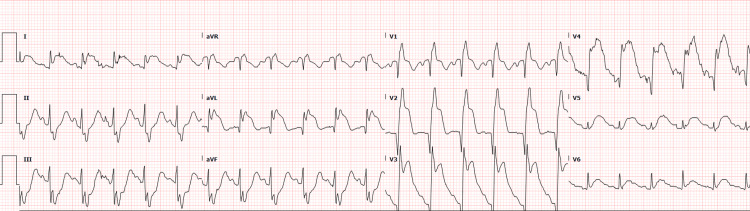
ECG showing anteroseptal myocardial infarction.

A 30-year-old male with a known diagnosis of Seckel syndrome, weighing only 11.5 kg, presented to a local clinic with severe chest and back pain. His past medical history was significant for seizure disorder, hypertension, chronic kidney disease, and a ruptured left posterior communicating artery aneurysm treated with endovascular coiling. An electrocardiogram performed at the clinic revealed an anteroseptal STEMI (Figure 1). He was administered aspirin 162 mg and clopidogrel 75 mg and was urgently transferred to our emergency department (ED) for further evaluation and management.

**Figure 2:**
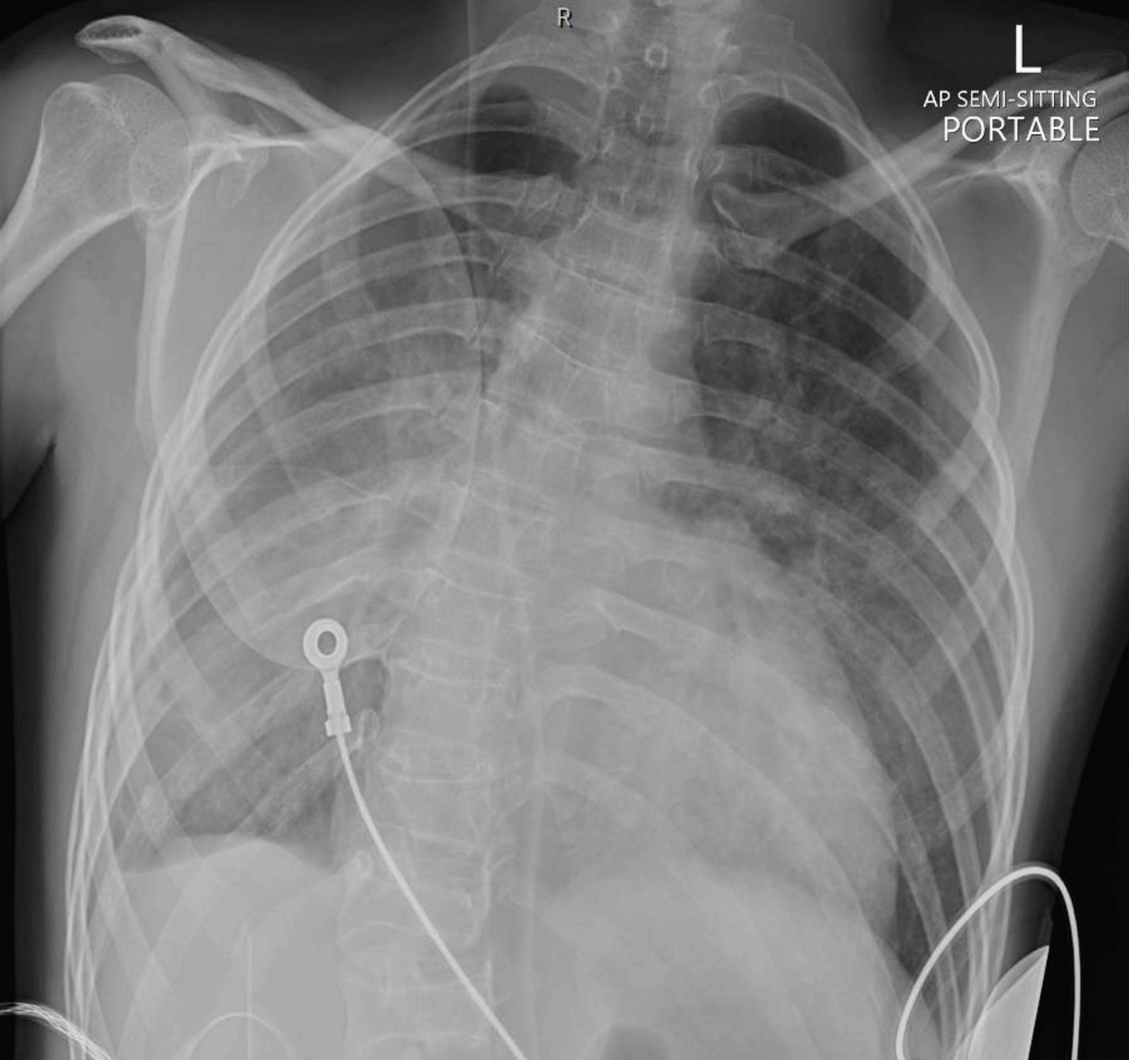
Chest X-ray showing signs of cardiogenic pulmonary edema. Bilateral prominent bronchovascular markings with perihilar infiltrates and blunting of the right costophrenic angle are noted, consistent with pulmonary vascular congestion and fluid overload in the context of acute heart failure.

Upon arrival, the patient was tachycardic, hypoxic, and in evident respiratory distress. Chest auscultation revealed bilateral crackles, and a chest radiograph confirmed the presence of cardiogenic pulmonary edema (Figure 2). Given his extremely low body weight, a weight-adjusted dose of 500 units of unfractionated heparin was administered. Due to impending respiratory failure and the anticipated need for hemodynamic stability during intervention, he was intubated in the ED by the anesthesiology team and started on inotropic support. Following initial stabilization, the patient was taken for emergent cardiac catheterization.

**Figure 3:**
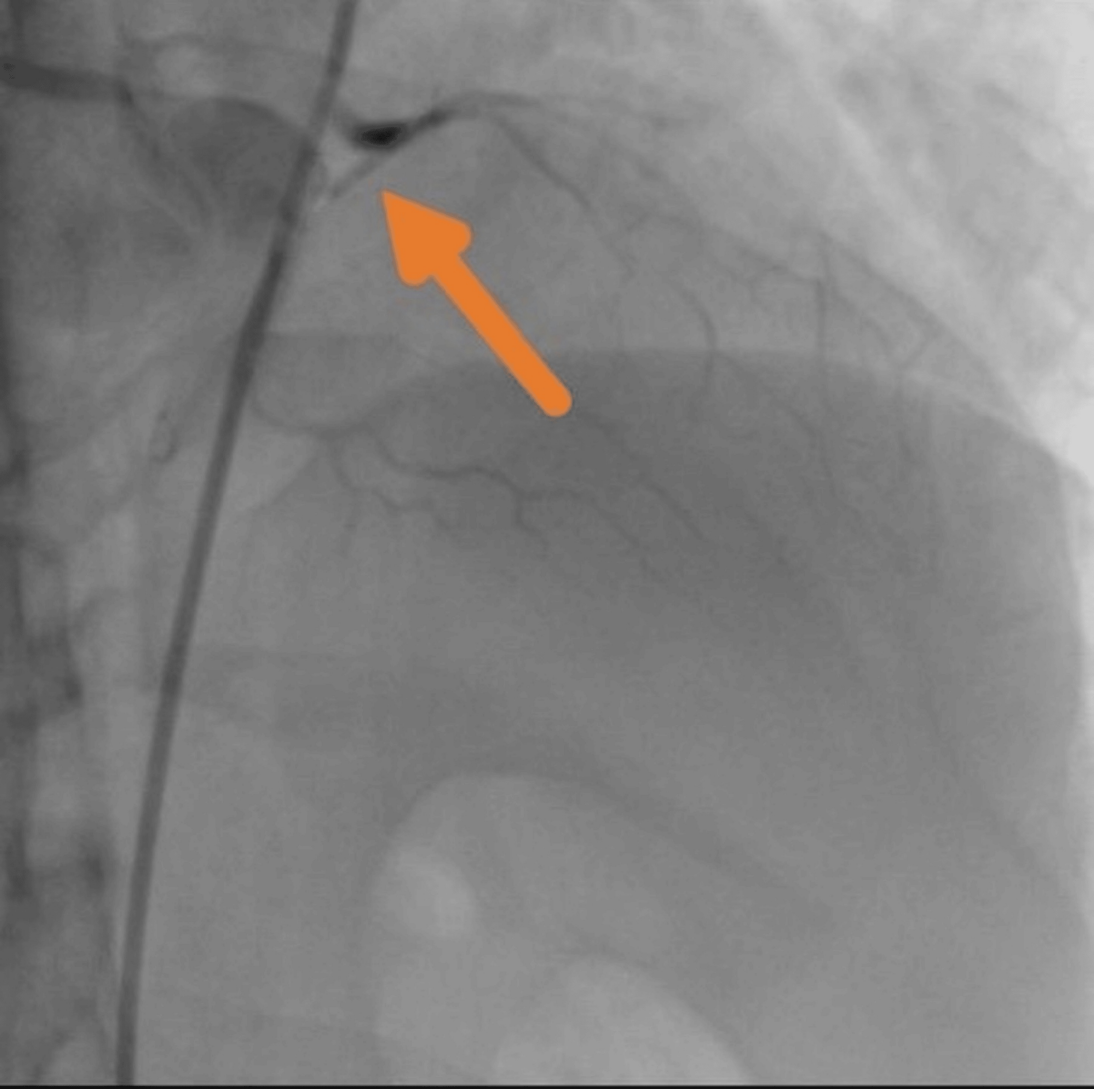
Coronary angiography revealing complete occlusion of the proximal LAD (arrow). Coronary angiogram demonstrates a 100% thrombotic occlusion of the proximal left anterior descending (LAD) artery with no flow beyond the lesion. The remainder of the coronary anatomy appears angiographically normal.

Coronary angiography demonstrated a 100% occlusion of the proximal left anterior descending (LAD) artery, with otherwise normal coronary anatomy (Figure 3). Due to the patient's diminutive size, no pediatric-sized guiding catheters were available nationally, and the use of standard adult-sized equipment was deemed unsafe. As a result, percutaneous coronary intervention (PCI) could not be performed. Additionally, given his prior history of a coiled intracranial aneurysm, thrombolytic therapy was contraindicated. Extensive consultations with tertiary cardiac centers across the UAE were initiated; however, none were able to offer pediatric coronary intervention due to a lack of appropriate equipment or procedural expertise. A multidisciplinary decision was therefore made to proceed with conservative medical management, and the patient was admitted to the adult intensive care unit (ICU).

Within 24 hours, the patient developed cardiorenal syndrome, became anuric, and required initiation of continuous renal replacement therapy (CRRT). Owing to his low body weight and multisystem complexity, he was subsequently transferred to the pediatric intensive care unit (PICU), where coordinated care was provided by pediatric intensive care, adult cardiology, nephrology, and critical care teams.

Laboratory investigations supported a diagnosis of cardiorenal syndrome and systemic hypoperfusion (Table 1). Brain natriuretic peptide (BNP) was markedly elevated at >35,000 ng/L (reference: 0.0-85.8 ng/L), and high-sensitivity troponin T exceeded 10,000 ng/L (reference: ≤14 ng/L), reflecting significant myocardial injury. Renal function deteriorated, with serum creatinine rising from a baseline of 127 µmol/L to 268 µmol/L, and estimated glomerular filtration rate (eGFR) declining from 65 to 26 mL/min/1.73m^2^. Serum lactate was elevated at 4.0 mmol/L (reference: 0.5-2.2 mmol/L), consistent with global hypoperfusion.

**Table 1:** Laboratory investigations on admission with corresponding reference ranges WBC: white blood cells; RBC: red blood cells; HCT: hematocrit; MCV: mean corpuscular volume; MCH: mean corpuscular hemoglobin; MCHC: mean corpuscular hemoglobin concentration; RDW-CV: red cell distribution width-coefficient of variation; AGAP: anion gap; NT-proBNP: N-terminal pro-B-type natriuretic peptide; eGFR: estimated glomerular filtration rate; CKD-EPI: chronic kidney disease epidemiology collaboration equation

Test	Result	Normal range
WBC (×10^9^/L)	34.9	4.0-11.0
RBC (×10^12^/L)	3.6	4.7-6.1 (male)
Hemoglobin (g/L)	104	135-175 (male)
HCT	0.31	0.40-0.52 (male)
MCV (fL)	85.3	80-100
MCH (pg)	28.9	27-33
MCHC (g/L)	339	320-360
Platelets (×10^9^/L)	62	150-400
RDW-CV (%)	18.3	11.5-14.5
Sodium (mmol/L)	138	135-145
Potassium (mmol/L)	5.3	3.5-5.1
Chloride (mmol/L)	103	98-107
CO2 (mmol/L)	16	22-29
AGAP (mmol/L)	19	8-16
Creatinine (µmol/L)	268	60-110 (male)
Urea (mmol/L)	21.8	2.5-7.8
Troponin T (ng/L)	>10,000	≤14
NT-pro BNP (ng/L)	>35,000	0.0-85.8
eGFR (CKD-EPI; mL/min/1.73m^2^)	26	>60

Despite aggressive management, including broad-spectrum antibiotics, CRRT, vasopressor support, and mechanical ventilation, his clinical condition deteriorated further, with progression to septic shock, refractory metabolic acidosis, and worsening multiorgan failure. The patient suffered a cardiac arrest in the PICU. Despite prolonged and extensive resuscitative efforts, he unfortunately did not survive.

## Discussion

This case represents the first documented instance of STEMI in a patient with Seckel syndrome, expanding the known cardiovascular phenotype of this rare primordial dwarfism syndrome. Prior reports have highlighted structural anomalies such as dilated cardiomyopathy and ventricular septal defects [2], as well as conduction abnormalities like sinus bradycardia and complete heart block [3]. Additionally, vascular anomalies such as intracranial aneurysms have been described, raising concerns about cerebrovascular fragility [6,7]. However, acute ischemic coronary events have not been recognized in this population, leaving a critical gap in clinical understanding.

The pathophysiologic mechanism of STEMI in this patient remains unclear, with possible contributors including accelerated atherosclerosis, microvascular dysfunction, or prothrombotic states inherent to Seckel syndrome. Similar vascular fragility and early arterial disease are reported in other primordial dwarfism syndromes, particularly MOPD II, where moyamoya vasculopathy [4,8], systemic arterial stenosis, and premature coronary disease are common [4]. His history of hypertension and chronic kidney disease likely further increased his risk of acute coronary syndrome. Mutations in ATR and other DNA damage response genes, commonly implicated in Seckel syndrome, may also predispose to vascular fragility and premature atherothrombosis, although this remains speculative [3,9].

According to the 2025 American College of Cardiology/American Heart Association (ACC/AHA) Joint Committee guideline for the management of STEMI, primary PCI remains the preferred reperfusion strategy, with fibrinolytic therapy recommended only when PCI cannot be performed within the advised time window [10]. In this case, however, both modalities were contraindicated: PCI due to anatomical inaccessibility and the unavailability of pediatric-compatible equipment, and thrombolysis due to a history of a coiled intracranial aneurysm. This reflects an exceptional clinical scenario where standard reperfusion pathways could not be applied, necessitating individualized conservative medical management based on multidisciplinary consensus. From a preventive standpoint, high-risk patients with Seckel or related syndromes may benefit from earlier cardiovascular surveillance, including regular echocardiographic screening and strict blood pressure control, as recommended in other primordial dwarfism syndromes [4].

As more patients with childhood-onset syndromes like Seckel syndrome reach adulthood, many fall outside the scope of standard adult interventional tools and protocols. A 2024 Journal of the American College of Cardiology (JACC) review emphasized the urgent need for integrated care pathways and hybrid catheterization laboratories tailored to morphologically pediatric adults [11]. This case underscores the necessity of expanding specialized pediatric interventional cardiology services, which remain limited in many regions, including the UAE.

Future studies and case series are required to better characterize cardiovascular risks in Seckel syndrome and to guide the development of individualized management strategies for morphologically pediatric adults with syndromic conditions.

## Conclusions

STEMI can occur in patients with Seckel syndrome and presents extraordinary challenges in diagnosis, access, and intervention. This case highlights the importance of developing specialized cardiovascular care pathways for morphologically pediatric adults and underscores the pressing need to expand pediatric interventional cardiology infrastructure in regions with growing adult congenital and syndromic populations.
